# Discourteous Treatment of an Expert Witness

**Published:** 1882-01

**Authors:** 


Discourteous Treatment of an Expert Witness.—Students of science everywhere cannot fail to notice with marked disapproval the efforts made by the prosecution in the great trial now going on at Washington, to depreciate the study of natural history, by applying the term "horse-doctor" to one of the distinguished expert witnesses, a well-known comparative anatomist and neurologist. It is evident that the testimony of this expert must have been regarded as of great weight, or the District Attorney would not have resorted to so unworthy a device to weaken it. We frankly admit that we are not in full accord with the testimony of said expert, but we consider that it shows the extensive knowledge this, so-called, veterinarian possesses of the diseases of the human brain. If it can be ascertained that such erudition has been derived from the study of veterinary subjects, we would suggest that the Court recommend some of the other experts to turn their attention to the study of the lower animals. We refer especially to those who do not believe in the transmission of diseases from parents to offspring. They would not, we are quite sure, succeed as stock raisers if they insisted on practising those theories

